# Editorial: Emerging techniques in Arabic natural language processing

**DOI:** 10.3389/frai.2025.1715520

**Published:** 2025-11-06

**Authors:** Shadi Abudalfa, Motaz Saad, Samhaa El-Beltagy

**Affiliations:** 1 SDAIA-KFUPM JRC for Artificial Intelligence, King Fahd University of Petroleum & Minerals, Dhahran, Saudi Arabia; 2 Department of Data Science, Islamic University of Gaza, Gaza, Palestine; 3 School of Information Technology, Newgiza University, Giza, Egypt

**Keywords:** Arabic NLP, detection, recognition, opinion mining, syntactic analyzers, machine translation, LLMS

## Introduction

Arabic Natural Language Processing (NLP) is a rapidly growing field focusing on the unique computational and linguistic challenges of the Arabic language. Recent progress has been driven by deep learning approaches and the increasing use of large language models (LLMs), which have improved applications such as sentiment analysis, text processing, speech recognition, and machine translation (Haboussi et al., 2025; Abdu et al., 2025). Despite these advances, the field still faces critical obstacles, including a shortage of annotated datasets, insufficient tools for dialect handling, and the limited availability of Arabic-oriented LLMs (Mashaabi et al., 2024; Dahou et al., 2025; Abudalfa et al., 2024). This Research Topic presents studies covering various aspects of Arabic NLP, such as syntactic analysis, dialect identification, stance classification, and other tasks that contribute to practical real-world solutions.

## Key contributions

The studies featured in this Research Topic highlight advancements in Arabic NLP and introduce innovative approaches within this field. The following subsections provide a concise overview of each paper included.

### Syntactic analyzers

Syntactic analysis is a core task in NLP, particularly vital for morphologically rich languages like Arabic. Saadiyeh et al. compared a range of Arabic syntactic analyzers, from rule-based, statistical, and machine learning approaches to hybrid, neural, and transformer-based models, examining their strengths, weaknesses, and trade-offs. The complexity of Arabic morphology and syntax makes accurate parsing challenging, which they address through a detailed evaluation of algorithms and their reliance on high-quality annotated resources.

### Machine translation

Algaraady and Mahyoob conducted a study comparing Arabic translations of Google Translate after post-editing by two professional translators and ChatGPT-4o, with three experts evaluating the final output. Quality was assessed through fluency, accuracy, coherence and efficiency, and a paired *t*-test analyzed the differences. Human post-editing generally yielded superior quality, while ChatGPT-4o stood out for speed and produced fluently flowing coherent translations.

In a related line of research, Beidas et al. examine the performance of GPT-3.5, GPT-4, and Bard (Gemini) on the QADI and MADAR datasets, whereas GPT-5 was tested solely on MADAR, which covers data from more than 15 countries. The evaluation relied on several metrics, including cosine similarity, the universal similarity encoder, sentence-BERT, TER, ROUGE, and BLEU. Two prompting strategies were applied: zero-shot and few-shot.

### Opinion mining

Alkhathlan et al. presented ArabicStanceX, a large dataset for stance detection with 14,477 tweets covering 17 topics. Using the transformer-based MARBERTv2 model and a Multi-Topic Single Model approach, they achieved an F1 score of 0.74 for “favor” and “against” categories and 0.67 overall. Their results reveal strengths in stance classification but also difficulties with neutral labels and unseen topics. Additional zero-shot and few-shot learning tests show the model's flexibility in adapting to new subjects.

Jaber et al. explored the use of ensemble-based machine learning approaches for Arabic sentiment classification. A range of homogeneous ensemble models is developed and tested on two corpora: the balanced ArTwitter dataset and the highly skewed Syria_Tweets dataset. To address the imbalance problem, the Synthetic Minority Over-sampling Technique (SMOTE) is applied. The experiments combine unigram features with pre-trained word embedding representations.

### Arabic poetry

Mutawa and Alrumaih presented a deep learning technique for identifying the meter of Arabic poetry using a large annotated dataset. Text was encoded at the character level to classify full and half verses without removing diacritics, ensuring that essential linguistic features were preserved. Various neural network architectures, including LSTM, GRU, and Bi-LSTM, were explored. This framework demonstrates a robust approach to Arabic meter recognition and highlights the potential of AI in NLP.

### Dialect detection

Saleh et al. presented a stacking-based technique to improve dialectal Arabic classification by combining two transformer models, Bert-Base-Arabertv02 and Dialectal-Arabic-XLM-R-Base. The technique involves two layers: the first generates class probabilities from the transformers, which are then used by a meta-learner in the second layer. This technique was benchmarked against individual models such as LSTM, GRU, CNN, and single transformers with various embeddings. Experimental results demonstrated that the combined model outperforms single-model methods by capturing a wider range of linguistic features, improving generalization across Arabic varieties.

### Speech recognition

Al-Anzi and Thankaleela presented an Arabic speech recognition framework that begins by extracting Mel-frequency cepstral coefficients (MFCCs) from audio signals. These features are then grouped through K-means clustering, and the resulting clusters are classified using methods such as Decision Trees, Random Forests, K-Nearest Neighbors, and XGBoost. For demonstration purposes, both Euclidean Distance and Dynamic Time Warping (DTW) are employed. Additionally, the research highlights the effectiveness of Mozilla's DeepSpeech framework in handling Arabic speech recognition.

### Cyberbullying detection

Allwaibed et al. reviewed 35 scholarly articles addressing the detection of cyberbullying in Arabic-language texts. From a methodological standpoint, traditional machine learning approaches that leverage Arabic-specific linguistic features continue to perform well on smaller datasets. However, more advanced deep learning models and transformer-based frameworks such as AraBERT achieve stronger results, especially when challenges like dialectal variation and orthographic inconsistencies are reduced.

## Conclusion

The studies gathered in this Research Topic illustrate the diversity and dynamism of ongoing efforts in Arabic NLP. Collectively, these contributions showcase how deep learning and LLMs are driving progress in Arabic NLP, while also pointing to persistent obstacles such as dialectal differences, scarcity of annotated data, and specialized domain challenges. By introducing innovative approaches, releasing new datasets, and offering comparative assessments, the featured works not only push the field forward but also stress the importance of sustained collaboration, resource creation, and tool development to enhance Arabic NLP and extend its practical impact.
